# Efficacy of DMARDs Therapy on the Disease Activity and Gene Expression Levels of FoxO1, FoxO3, Runx1, and Runx3 in Early Rheumatoid Arthritis Patients: A Pre–Post Interventional Study With Healthy Controls

**DOI:** 10.1002/hsr2.71388

**Published:** 2025-10-21

**Authors:** Seyed Askar Roghani, Ramin Lotfi, Shirin Assar, Mahdi Taghadosi, Seyedeh Zahra Shahrokhvand, Ghazal Hosseini Torshizi, Mehran Pournazari, Parviz Soufivand, Afsaneh Shamsi, Bahareh Kardideh, Fatemeh Khademi, Alireza Nasirifar

**Affiliations:** ^1^ Department of Anatomical Sciences, Faculty of Medical Sciences Tarbiat Modares University Tehran Iran; ^2^ Blood Transfusion Research Center, High Institute for Research and Education in Transfusion Medicine Tehran Iran; ^3^ Clinical Research Development Center, Tohid Hospital Kurdistan University of Medical Sciences Sanandaj Iran; ^4^ Clinical Research Development Center, Imam Reza Hospital Kermanshah University of Medical Sciences Kermanshah Iran; ^5^ Cardiovascular Research Center, Health Technology Institute Kermanshah University of Medical Sciences Kermanshah Iran; ^6^ Department of Biology, Mashhad Branch Islamic Azad University Mashhad Iran; ^7^ Medical Biology Research Center, Health Technology Institute Kermanshah University of Medical Sciences Kermanshah Iran; ^8^ Department of Internal Medicine, School of Medicine Kurdistan University of Medical Sciences Sanandaj Iran

**Keywords:** disease activity, forkhead box O3, interleukin‐6, rheumatoid arthritis, Runx3

## Abstract

**Background and Aims:**

Forkhead box O (FoxO) transcription factors exert crucial roles in immune responses. Besides, the Runt‐related transcription factor (Runx) family plays a pivotal role in the development and homeostasis of cartilage and bone. This study assessed the plasma concentrations of the pro‐inflammatory interleukin‐6 (IL‐6) cytokine and the anti‐inflammatory cytokine interleukin‐10 (IL‐10), as well as the gene expression levels of FoxO1, FoxO3, Runx1, and Runx3, in the peripheral blood sample of the early rheumatoid arthritis (RA) patients treated with conventional disease‐modifying antirheumatic drugs (DMARDs) for 6 months relative to normal subjects.

**Methods:**

This study examined 30 early DMARDs‐naïve RA patients (before and after treatment) and 30 age‐ and gender‐matched normal individuals. The plasma levels of IL‐6 and IL‐10 were measured by enzyme‐linked immunosorbent assay (ELISA), and the mRNA expression amounts of FoxO1, FoxO3, Runx1, and Runx3 were determined using real‐time PCR.

**Results:**

The plasma IL‐6 concentrations increased significantly in pre‐ and posttreatment RA patients compared with controls (*p* < 0.001). Moreover, the gene expression of FoxO1 and FoxO3 was enhanced considerably in RA patients (both before and after treatment), compared to controls (*p* < 0.001 for FoxO1 and *p* = 0.02 and *p* = 0.01 for FoxO3, respectively). Runx1 gene expression increased dramatically in pre‐ and posttreatment RA patients compared with the controls (*p* < 0.001). Runx3 gene expression was meaningfully enhanced in pretreatment RA patients than in normal controls (*p* < 0.001). Also, DMARDs treatment strikingly reduced Runx3 gene expression levels compared to pretreatment levels (*p* < 0.001).

**Conclusion:**

DMARDs treatment dramatically diminishes Runx3 gene expression, thereby lowering disease activity and inflammatory markers in the early RA patients.

**Abbreviations**
Anti‐CCP: Anti‐cyclic citrullinated peptide
CRP: C‐reactive protein
DAS28: Disease Activity Score 28
DMARDs: disease‐modifying antirheumatic drugs
ELISA: enzyme‐linked immunosorbent assay
ESR: erythrocyte sedimentation rate
FoxO3: forkhead box O3
IL‐6: interleukin‐6
RA: rheumatoid arthritis
Runx: runt‐related transcription factor
Treg cell: regulatory T cell

## 1 Introduction

Rheumatoid arthritis (RA) is a systemic, progressive, inflammatory autoimmune disorder that symmetrically affects the small joints of the person. RA affects almost 1% of the world population and is characterized by chronic inflammation of the synovial joints, leading to progressive joint injury, bone destruction, and disability [1, 2, 3]. Disease‐modifying antirheumatic drugs (DMARDs) are immunosuppressive and immunomodulatory medicines used to treat autoimmune disorders, like RA. As the first‐line therapy for RA, DMARDs help preserve joints by suppressing inflammation, diminishing disease activity, inducing remission, and stopping or slowing down the progression of inflammatory forms of arthritis [4].

Forkhead box O (FOXO) transcription factors, belonging to the forkhead box family of transcription factors, are characterized structurally by an evolutionarily conserved winged‐helix DNA binding domain with ~100 amino acids in length. This subfamily regulates multiple cellular processes, like cell cycle, apoptosis, and metabolism, and also consists of four members, including FoxO1, FoxO3 (also known as FoxO3a), FoxO4, and FoxO6. Of note, FoxO1 and FoxO3 are the major isoforms expressed in the immune system [5, 6]. In this context, FoxO1 is a downstream molecule of the phosphatidylinositol 3‐kinase (PI3K)‐AKT signaling pathway, resulting in the production of interleukin‐10 (IL‐10) in B cells [7]. FoxO1 has also been shown to control the development and function of regulatory (Treg) T cells [8]. FoxO1 gene expression levels were lower in RA patients' peripheral blood compared with controls, and the expression of FoxO1 in RA synovial tissue was negatively associated with illness activity [9]. Besides, FoxO3 is known to regulate oxidative stress resistance, innate immune homeostasis, immune responses, and inflammation [10]. According to evidence, the expression of FoxO3 was increased in RA patients compared with the controls [11, 12]. Also, FoxO3 expression was positively associated with erythrocyte sedimentation rate (ESR), C‐reactive protein (CRP), and Disease Activity Score 28 (DAS28) of RA patients [12].

The Runt‐related transcription factor (Runx) family is an evolutionarily conserved family of transcription factors with three members: Runx1/AML1, Runx2, and Runx3. This family exerts critical roles in the development and homeostasis of cartilage and bone and also regulates the expression of genes implicated in embryonic development and cellular differentiation [13, 14]. Single‐nucleotide polymorphisms (SNPs) in the *Runx1* gene were found to be associated with autoimmune disorders, such as RA [15]. Moreover, it has been shown that overexpression of Runx1 strikingly inhibits the invasive phenotype of RA‐associated fibroblast‐like synoviocytes (RA‐FLS) and suppresses the expression of pro‐inflammatory cytokines IL‐6 and IL‐1β [16]. Notably, Runx3 has been reported to protect articular cartilage against posttraumatic osteoarthritis [13]. Given the above‐mentioned explanations, the current study intended to assess the effectiveness of DMARDs therapy on the illness activity and gene expression levels of FoxO1, FoxO3, Runx1, and Runx3 in early RA patients, in comparison with the normal subjects. Also, the levels of IL‐6 and IL‐10 were evaluated in the plasma of RA patients and controls.

## 2 Materials and Methods

### 2.1 Studied Individuals

This study employed a pre–post interventional design with a parallel healthy control group. A total of 30 early DMARDs‐naïve RA patients (before and after treatment) were recruited from Imam Reza Hospital, which is located in Kermanshah province, Iran. All the studied patients were diagnosed with RA by an expert rheumatologist according to the American College of Rheumatology/European League Against Rheumatism (ACR/EULAR) 2010 classification criteria for RA [17]. Notably, all patients received conventional DMARDs therapy (Methotrexate [7.5–15 mg/day], Hydroxychloroquine [200 mg/day], and methylprednisolone [mPRED: 5–15 mg/day]) for 6 months. All drug doses were administered within the standard therapeutic ranges recommended by EULAR and were further adjusted individually based on each patient's DAS28 score and clinical response. To strengthen the design, 30 age‐ and sex‐matched healthy individuals without a history of autoimmune disease were included as a control group.

### 2.2 Ethics Statement

This study complied with the Declaration of Helsinki and was approved by the Kermanshah University of Medical Sciences (KUMS) ethical committee (Ethical code: IR.KUMS.REC.1399.403). All the patients and normal people signed the written informed consent form.

### 2.3 Blood Specimen Collection

Ten milliliters (10 mL) of peripheral blood specimen was obtained from each participant. After that, plasma specimens were instantly isolated from 8 mL of the collected blood specimen for measurement of the pro‐inflammatory IL‐6 and anti‐inflammatory IL‐10 cytokines. For the extraction of the total RNA, 2 mL of the remaining blood sample was aliquoted into the tubes containing ethylenediamine tetraacetic acid (EDTA) as an anticoagulant.

### 2.4 Measuring the Plasma Levels for IL‐6 and IL‐10 Cytokines

By using the enzyme‐linked immunosorbent assay (ELISA) kits (Demeditec, Germany, and KPG, Iran, in order), the plasma levels of IL‐6 and IL‐10 cytokines were quantified following the manufacturer's guidelines.

### 2.5 RNA Isolation, cDNA Synthesis, and Real‐Time PCR

Total RNA was extracted from peripheral blood samples using the RNA extraction kit (RNX PLUS, Sina Clon, Iran) based on the instructions of the manufacturer. The concentration and purity of the extracted total RNA were determined by the Nanodrop 2000 UV‐vis spectrophotometer (Thermo Scientific, the United States). After that, the reverse transcription of the extracted RNA into complementary DNA (cDNA) was done using the cDNA synthesis kit (Roch, Switzerland) according to the manufacturer's guidelines. All samples were finally kept in a −70°C freezer until use. Primers for the investigated genes of FoxO1, FoxO3, Runx1, Runx3, and glyceraldehyde 3‐phosphate dehydrogenase (GAPDH), as an internal control for normalization, were designed by using online websites, including UCSC, Oligocalc, and Oligoanalyzer. The nucleotide sequences of the used primers and their PCR product size are shown in Table 1. In terms of their accuracy and specificity, the designed primers were initially checked and then verified by using the Basic Local Alignment Search Tool on the US National Center for Biotechnology Information (NCBI) website (http://www.ncbi.nlm.nih.gov/tools/primerblast/). Quantitative real‐time PCR was done in a final volume of 15 µL encompassing 1 μL of cDNA, 0.5 μL of each forward and reverse primer, 7.5 μL of PCR Master Mix (Parstous Biotechnology), and 5.5 μL of ddH2O. The PCR was accomplished on the Light cycler 96 instrument (Roche) by the thermal cycling parameters (30 s at 95°C, 40 cycles of 5 s at 95°C, 30 s at 60°C, 61°C, 62°C, 62°C, and 65°C for GAPDH, FoxO1, FoxO3, Runx1, and Runx3, in order; melting curve: 5 s at 95°C, 15 s at 65°C, 5 s at 95°C, and continues melting). All specimens were run in duplicates to reduce the risk of technical errors causing false‐positive/negative results. The relative amount of the investigated target mRNAs in samples was computed and normalized to the corresponding GAPDH mRNA transcript level as a housekeeping reference gene. The relative expression of genes for each sample to the internal reference gene was assessed using the following formula (ratio = (E_target_)^ΔCt target (control‐sample)^/(E_Ref_)^ΔCt Ref (control‐sample)^), as previously demonstrated by Pfaffl [18].

**Table 1:** The designed primer sequences of the investigated target and reference genes for real‐time PCR amplification.

Gene	Forward primer (5′ > 3′)	Reverse primer (5′ > 3′)	PCR product size (bp)
*GAPDH*	GAAACCTGCCAAGTATGATG	AGGAAATGAGCTTGACAAAG	188
*FoxO1*	TGGACATGCTCAGCAGACATC	TTGGGTCAGGCGGTTCATAC	97
*FoxO3*	CCCAGCCTAACCAGGGAAGT	AGCGCCCTGGGTTTGG	68
*Runx1*	TCAGGTTTGTCGGTCGAAG	GCCCATCCACTGTGATTTTG	117
*Runx3*	CAGAAGCTGGAGGACCAGAC	GTCGGAGAATGGGTTCAGTT	180

### 2.6 Statistics

Data analysis was accomplished using the SPSS software (version 21.0, SPSS Inc.) and the software GraphPad Prism 6.0 (GraphPad Software). At first, the normality of the data was determined by the 1‐sample Kolmogorov–Smirnov (1‐sample K‐S) test. One‐way ANOVA test was applied to compare the data analysis between the three studied groups. Analysis of correlations between all the studied variables was performed using Spearman's and Pearson's rank correlations. No correction for multiple testing was applied. In all statistical analyses, data were represented as mean ± standard error of the mean (SEM), and a *p* value less than 0.05 was considered significant.

## 3 Results

### 3.1 Demographic and Clinical Characteristics of the Studied Individuals

The current study assessed 30 early RA patients treated with conventional DMARDs for 6 months and 30 normal individuals. Regarding age and gender variables, there was no significant difference between early RA patients and controls. Besides, therapy with DMARDs strikingly decreased the anti‐cyclic citrullinated peptide (anti‐CCP), ESR, Swollen, Tender, and DAS28, in comparison with the pretreatment RA patients (*p* < 0.001). The demographic information and clinical properties of the studied subjects are indicated in Table 2.

**Table 2:** Demographic information and clinical characteristics of the studied participants.

Variable	RA patients		Control	*p* value
	Before treatment	After treatment		
*N*	30	30	30	
Age (Y)	50.6±1.94	50.6±1.94	51.4 ± 1.64	
Gender (F/M)	22/8	22/8	22/8	
AntiCCP‐IgG (U/mL)	877.89 ± 107.6	298.64 ± 66.2	/	< 0.001
ESR (mm/h)	36.4 ± 3.08	20.2 ± 1.75	/	< 0.001
DAS‐28	4.12 ± 0.13	2.45 ± 0.14	/	< 0.001
Swollen	3.97 ± 0.49	2.2 ± 0.59	/	< 0.001
Tender	4.43 ± 0.50	2.3 ± 0.65	/	< 0.001
IL‐6 (pg/mL)	164.82 ± 6.42	143.11 ± 5.41	66.91 ± 3.11	< 0.001
IL‐10 (pg/mL)	176.95 ± 15.07	174.85 ± 11.01	159.27 ± 14.91	= 0.18

*Note:* All data were presented as the mean value ± SEM.

Abbreviations: DAS‐28, Disease Activity Score 28; ESR, erythrocyte sedimentation rate; *N*, number.

### 3.2 Plasma Levels of IL‐6 and IL‐10

**Figure 1:**
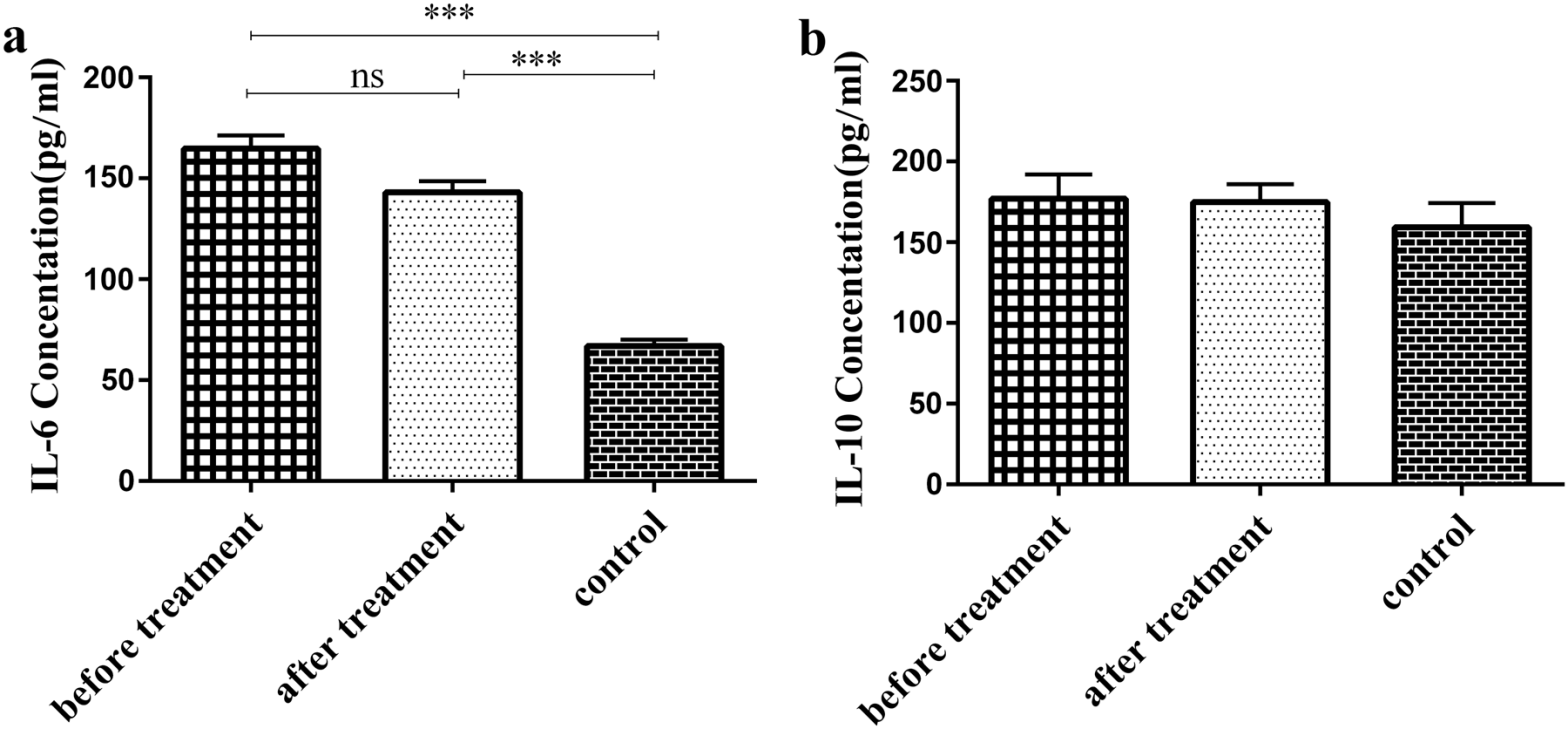
Plasma concentration analysis. (a) Plasma concentrations of IL‐6 in RA patients and the control group. IL‐6 plasma concentrations increased significantly in both pre‐ and posttreatment RA patients compared with controls (*p* < 0.001). (b) IL‐10 plasma levels in RA patients and controls. There was no meaningful difference among the three studied groups in terms of IL‐10 plasma levels. ns, not statistically significant; *** *p* < 0.001.

The results depicted that the mean plasma concentrations of IL‐6 were strikingly enhanced in pre‐ and posttreatment RA patients, in comparison with controls (*p* < 0.001). Following DMARDs treatment, plasma IL‐6 concentrations diminished, but not considerably, compared with pretreatment RA patients (Figure 1a and Table 2). In the case of IL‐10 plasma concentrations, there was no meaningful difference among the three studied groups (*p* = 0.18) (Figure 1b and Table 2).

### 3.3 Gene Expression Assessment of FoxO1, FoxO3, Runx1, and Runx3

**Figure 2:**
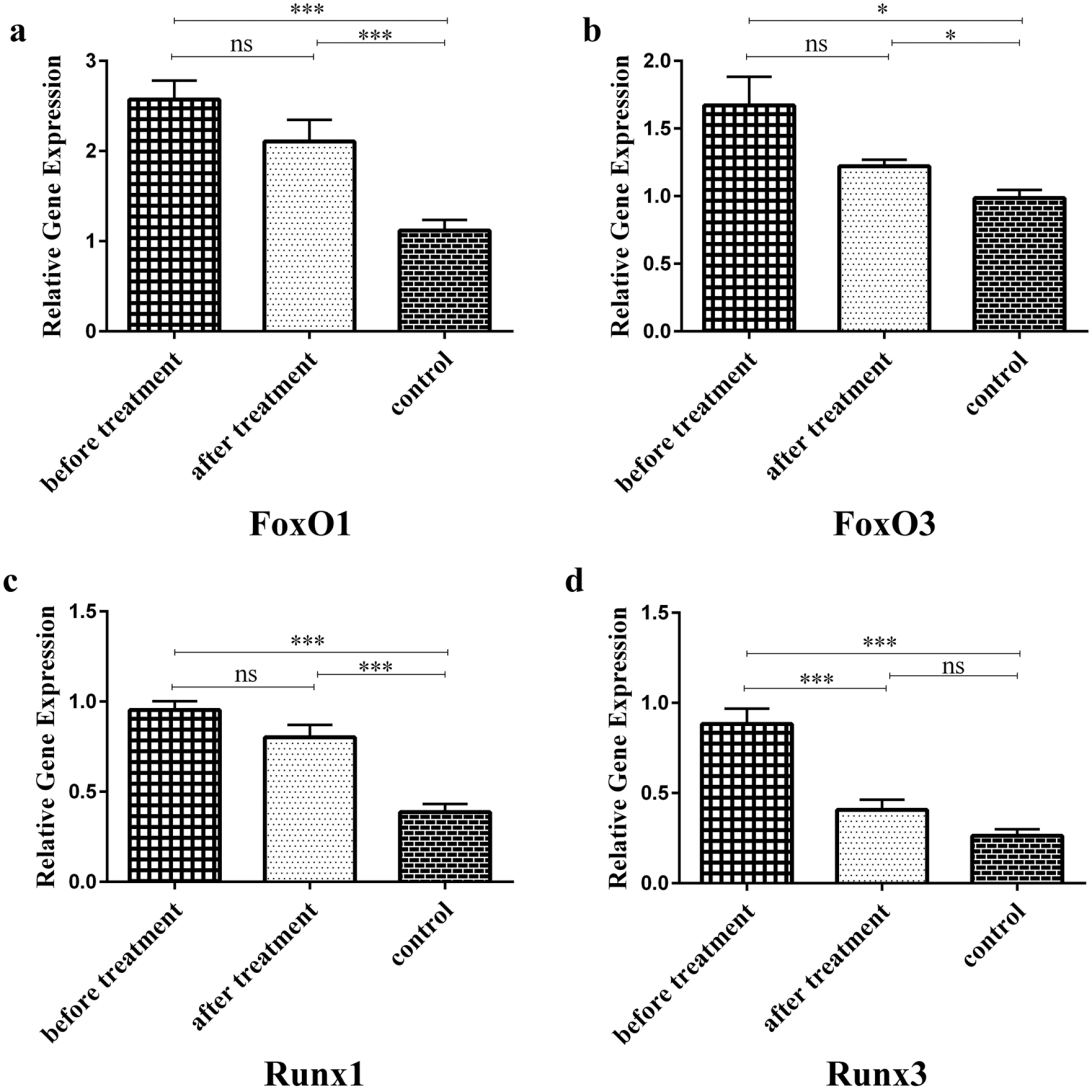
Gene expression level analysis. (a) Gene expression of FoxO1 in RA patients and controls. The gene expression level of FoxO1 was significantly enhanced in both pre‐ and posttreatment RA patients relative to controls (2.57 and 2.11 versus 1.12, *p* < 0.001). (b) Gene expression of FoxO3 in RA patients and controls. FoxO3 gene expression level increased meaningfully in both pre‐ and posttreatment RA patients than in controls (1.67 and 1.22 versus 0.98, *p* = 0.02 and *p* = 0.01, in order). (c) Runx1 gene expression level in patients and controls. The gene expression level of Runx1 was significantly higher in both pre‐ and posttreatment RA patients compared with controls (0.95 and 0.8 versus 0.39, *p* < 0.001). (d) The gene expression level of Runx3 in RA patients and controls. Runx3 gene expression was significantly greater in pretreatment RA patients than in controls (0.88 versus 0.26, *p* < 0.001). Besides, DMARDs therapy strikingly decreased the gene expression level of Runx3 relative to pretreatment RA patients (0.41 versus 0.88, *p* < 0.001). ns, not statistically significant; * *p* < 0.05; *** *p* < 0.001.

Gene expression level analysis indicated that the gene expression of FoxO1 and FoxO3 enhanced significantly in RA patients (both pre‐ and posttreatment), compared to healthy controls (2.57 and 2.11 versus 1.12, *p* < 0.001 for FoxO1 and 1.67 and 1.22 versus 0.98, *p* = 0.02 and *p* = 0.01 for FoxO3, respectively). After DMARDs therapy, FoxO1 and FoxO3 gene expression decreased relative to pretreatment RA patients; however, their difference was not statistically meaningful (Figure 2a and b). Furthermore, Runx1 gene expression was markedly increased in both the pre‐ and posttreatment RA patients, relative to the controls (0.95 and 0.8 versus 0.39, *p* < 0.001). In terms of Runx1 gene expression level, there was no statistically meaningful difference between pre‐ and posttreatment RA patients (Figure 2c). Runx3 gene expression was meaningfully enhanced in pretreatment RA patients than in controls (0.88 versus 0.26, *p* < 0.001). Also, DMARDs treatment dramatically reduced the gene expression levels of Runx3, compared to pretreatment levels (0.41 versus 0.88, *p* < 0.001) (Figure 2d).

### 3.4 Correlation Analysis

There existed positive correlations between anti‐CCP and FoxO1 (*r* = 0.330, *p* < 0.001), anti‐CCP and Runx1 (*r* = 0.476, *p* < 0.001), anti‐CCP and Runx3 (*r* = 0.554, *p* < 0.001), anti‐CCP and IL‐6 (*r* = 0.532, *p* < 0.001), anti‐CCP and IL‐10 (*r* = 0.331, *p* < 0.001), anti‐CCP and DAS‐28 (*r* = 0.507, *p* < 0.001), DAS28 and FoxO1 (*r* = 0.483, *p* < 0.001), DAS28 and Runx1 (*r* = 0.444, *p* < 0.001), DAS28 and Runx3 (*r* = 0.640, *p* < 0.001), DAS28 and IL‐6 (*r* = 0.389, *p* = 0.002), DAS28 and IL‐10 (*r* = 0.292, *p* = 0.02), IL‐6 and FoxO1 (*r* = 0.546, *p* < 0.001), IL‐6 and FoxO3 (*r* = 0.234, *p* = 0.03), IL‐6 and Runx1 (*r* = 0.407, *p* = 0.001), IL‐6 and Runx3 (*r* = 0.536, *p* < 0.001), IL‐10 and FoxO1 (*r* = 0.377, *p* = 0.003), IL‐10 and Runx1 (*r* = 0.405, *p* = 0.001), IL‐10 and Runx3 (*r* = 0.310, *p* = 0.02), IL‐10 and DAS28 (*r* = 0.292, *p* = 0.02), Runx1 and FoxO1 (*r* = 0.454, *p* < 0.001), Runx1 and Runx3 (*r* = 0.555, *p* < 0.001), Runx3 and FoxO1 (*r* = 0.341, *p* = 0.008), and FoxO1 and FoxO3 (*r* = 0.264, *p* = 0.012). The correlation analysis results between the investigated variables are illustrated in Table 3.

**Table 3:** Correlation analysis between investigated variables in RA patients.

		FoxO1	FoxO3	Runx1	Runx3	IL‐6	IL‐10	DAS28
Anti‐CCP	*p*	**< 0.001**	0.92	**< 0.001**	**< 0.001**	**< 0.001**	**< 0.001**	**< 0.001**
	*r*	0.330	0.013	0.476	0.554	0.532	0.331	0.507
DAS28	*p*	**< 0.001**	0.08	**< 0.001**	**< 0.001**	**0.002**	**0.02**	—
	*r*	0.483	0.563	0.444	0.640	0.389	0.292	—
IL‐6	*p*	**< 0.001**	**0.03**	**0.001**	**< 0.001**	—	0.05	**0.002**
	*r*	0.546	0.234	0.407	0.536	—	0.310	0.389
IL‐10	*p*	**0.003**	0.22	**0.001**	**0.02**	0.05	—	**0.02**
	*r*	0.377	0.131	0.405	0.310	0.310	—	0.292
Runx1	*p*	**< 0.001**	0.06	—	**< 0.001**	**0.001**	**0.001**	**< 0.001**
	*r*	0.454	0.199	—	0.555	0.407	0.405	0.444
Runx3	*p*	**0.008**	0.07	**< 0.001**	—	**< 0.001**	**0.02**	**< 0.001**
	*r*	0.341	0.194	0.555	—	0.536	0.310	0.640
FoxO1	*p*	—	**0.012**	**< 0.001**	**0.008**	**< 0.001**	**0.003**	**< 0.001**
	*r*	—	0.264	0.454	0.341	0.546	0.377	0.483
FoxO3	*p*	**0.012**	—	0.06	0.07	**0.03**	0.22	0.08
	*r*	0.264	—	0.199	0.194	0.234	0.131	0.563

*Note:* Significant data are presented in bold.

## 4 Discussion

RA is a chronic inflammatory autoimmune rheumatic disease characterized by the synovial influx of immune cells leading to progressive joint destruction [2]. In this study, we evaluated the plasma concentrations of IL‐6 and IL‐10 cytokines, as well as the gene expression levels of FoxO1, FoxO3, Runx1, and Runx3, in the peripheral blood specimens of early RA patients who were treated with conventional DMARDs for 6 months, in comparison with healthy individuals.

In our study, patients received conventional DMARDs including methotrexate, hydroxychloroquine, and low‐dose methylprednisolone. While all are categorized as conventional DMARDs, their mechanisms of action are not identical. Methotrexate primarily acts as a folate antagonist with immunosuppressive effects on T‐cell activation and pro‐inflammatory cytokines [19]. Hydroxychloroquine functions as an immunomodulator by interfering with lysosomal antigen processing and Toll‐like receptor signaling [20]. In contrast, corticosteroids such as methylprednisolone exert rapid and broad suppression of inflammatory gene transcription through glucocorticoid receptor signaling [21]. These drugs, therefore, exert distinct but partially overlapping immunological effects. Nevertheless, the combination of these agents is consistent with international treatment recommendations for early RA, where methotrexate is considered the cornerstone of therapy, often complemented by other DMARDs or low‐dose glucocorticoids to optimize treatment outcomes [22, 23].

IL‐6 is well known as a pro‐inflammatory cytokine, exerting a critical role in various aspects of systemic inflammation and RA pathogenesis, and it is also associated with joint destruction and illness activity [24]. Moreover, IL‐6 can be regarded as an indicator in assessing the severity of illness activity and is linked with an enhanced risk of developing RA‐associated comorbidity conditions, such as cardiovascular events [25, 26]. According to evidence, IL‐6 serum levels were significantly greater in RA patients than in controls, and higher IL‐6 levels were associated with swollen joints, limited functional capacity, and illness activity [25]. Compatible with this study's findings, IL‐6 plasma concentrations were recently found to be increased considerably in RA patients, compared with controls [24]. In the present study, the mean plasma concentrations of IL‐6 are significantly greater in pre‐ and posttreatment RA patients relative to controls, and IL‐6 plasma levels were positively correlated with anti‐CCP and DAS28.

IL‐10 is an anti‐inflammatory cytokine generated by Treg cells, accounting for the immunosuppressive and regulatory actions of these cells [27]. The present study demonstrated no significant difference in the plasma IL‐10 concentrations between RA patients and controls. In concordance with these findings, a recent study has illustrated that IL‐10 plasma levels are not considerably different between RA patients (newly diagnosed and undertreatment) and healthy controls [2].

FoxO transcription factors exert a critical role in regulating lymphocyte proliferation and activation [28]. The mRNA expression levels of FoxO1 were found to be strikingly lower in the peripheral blood of RA patients than in healthy controls, and FoxO1 expression in RA synovial tissue was negatively correlated with disease activity [9]. Similarly, another study indicated that the transcript levels of FoxO1 were significantly decreased in RA and systemic lupus erythematosus (SLE) patients compared with those in healthy controls, and FoxO1 transcript levels were negatively associated with lupus disease activity [28]. However, in the current study, the gene expression of FoxO1 increased significantly in RA patients (both pre‐ and posttreatment), compared with healthy controls. Moreover, following DMARDs treatment, FoxO1 gene expression decreased compared to pretreatment RA patients; yet, their difference was not statistically meaningful.

FoxO3 has been shown to regulate immunological homeostasis and tolerance by controlling the development and action of various immune cells. Evidence indicates that FoxO3 transcription levels are diminished in different autoimmune disorders and are intimately associated with the illness activity [29]. In comparison with healthy controls, the mRNA expression levels of FoxO3 in peripheral blood mononuclear cells (PBMCs) were not considerably changed in RA patients [11, 28], nor in whole blood [9], but strikingly reduced in CD14^+^ PBMCs [30]. Contrariwise, the mRNA and protein expression levels of FoxO3 in leukocytes were significantly enhanced, in particular, in polymorphonuclear cells (PMNs) [11]. Moreover, FoxO3 levels were significantly diminished in the synovial tissue of RA patients relative to controls [31, 32, 33]. However, in the present study, the gene expression levels of FoxO3 increased significantly in RA patients (both pre‐ and posttreatment), compared with controls. Furthermore, FoxO3 gene expression was positively associated with FoxO1 gene expression and IL‐6 plasma levels.

Altogether, in this study, FoxO1 and FoxO3 were upregulated in RA patients compared with healthy controls. This contrasts with some prior reports, likely reflecting differences in sample type, methodology, and patient characteristics. Our analysis used whole blood, integrating diverse immune cell subsets, whereas other studies focused on isolated populations such as T cells or synovial fibroblasts [9, 34]. We measured mRNA levels by qPCR, while others relied on proteomic or posttranslational approaches, which may yield different patterns [35]. Variability in disease stage, treatment exposure, and cohort heterogeneity also affects expression profiles [28]. Together, these factors suggest that the increased FoxO1 and FoxO3 expression observed here reflects the complex regulatory landscape of early RA and underscores the importance of methodological context in interpreting molecular data.

Runx proteins have been surveyed for a long time to act as pivotal regulators of cellular differentiation [14]. Recently, overexpression of Runx1 has been reported to considerably inhibit the invasive phenotype of RA‐FLS and suppress the expression of the pro‐inflammatory cytokines IL‐6 and IL‐1β [16]. In the present study, Runx1 gene expression was dramatically enhanced in both the pre‐ and posttreatment RA patients compared with the controls. Moreover, there was no statistically meaningful difference between pre‐ and posttreatment RA patients in terms of Runx1 gene expression level. Runx1 gene expression was also positively associated with DAS28, gene expression of FoxO1 and Runx3, and plasma concentrations of IL‐6 and IL‐10.

According to evidence, Runx3 not only inhibits the differentiation of Th2 cells but also enhances the expression of the effector molecules specific to T helper 1 (Th1) cells, such as interferon‐γ (IFN‐γ) [14, 36]. In this study, the gene expression of Runx3 was dramatically enhanced in pretreatment RA patients compared to controls. Also, DMARDs treatment markedly reduced the gene expression levels of Runx3, compared to pretreatment levels. Besides, Runx3 expression was positively associated with DAS28, gene expression of FoxO1 and Runx1, and plasma concentrations of IL‐6 and IL‐10. Since Th1 cells and their produced cytokines potentially contribute to the progression and pathogenesis of RA [37], DMARDs therapy‐mediated reduction of the gene expression of Runx3, which in turn decreases Th1 lineage differentiation, causes lower illness activity and inflammatory markers in the early RA patients.

The present study has several limitations. First, this study was conducted with a relatively small cohort of 30 patients and 30 healthy controls, mainly due to limited access to newly diagnosed, treatment‐naïve patients. Although this sample size is consistent with exploratory research in RA, it may have restricted the statistical power and the generalizability of the findings. Therefore, the results should be interpreted with caution. Future studies involving larger, multicenter populations and supported by pre‐specified power calculations are needed to confirm and extend these results. Second, synovial fluid samples were not available due to ethical considerations. Third, although patients were matched for age and sex, other potential confounders—including BMI, smoking status, and comorbidities—were not assessed. The absence of these data may have influenced systemic inflammation and gene expression and represents an important limitation that should be addressed in future studies. Fourth, treatment heterogeneity should be acknowledged. Although all patients received the same drug combination, doses were individually adjusted within standard therapeutic ranges. Because of the small sample size, stratified analyses by dosing regimen were not feasible and should be considered when interpreting the findings. Fifth, because no adjustment for multiple testing was applied, there remains a possibility of type I error. Accordingly, these findings should be considered exploratory and interpreted with caution. Finally, protein expression levels of FoxO1, FoxO3, Runx1, and Runx3 were not evaluated; incorporating both gene and protein analyses in future studies would provide deeper insights into the functional relevance of these transcription factors in RA.

## 5 Conclusion

DMARD therapy in early RA was associated with reduced Runx3 expression, paralleling improvements in disease activity and decreases in inflammatory markers. Larger studies with protein‐level validation are needed to confirm this association and clarify its clinical significance. Additionally, our findings show that plasma concentrations of IL‐6 and gene expression of FoxO1, FoxO3, Runx1, and Runx3 were dramatically greater in the early RA patients relative to healthy controls. Notably, Runx3 expression was positively associated with DAS28, gene expression of FoxO1 and Runx1, and plasma concentrations of IL‐6 and IL‐10, underscoring its potential role in the disease. Further studies are imperative in this area to corroborate our findings.

## Consent to Publish

The authors confirm that human research participants provided informed consent for the publication of the manuscript results.

## Author Contributions

**Fatemeh Khademi:** conceptualization, funding acquisition, project administration, supervision, validation, writing – review and editing. **Seyedeh Zahra Shahrokhvand:** formal analysis, software, writing – review and editing. **Ghazal Hosseini Torshizi:** formal analysis, software, writing – review and editing. **Bahareh Kardideh:** formal analysis, software, writing – review and editing. **Ramin Lotfi:** investigation, writing – original draft, writing – review and editing. **Seyed Askar Roghani:** investigation, writing – original draft, writing – review and editing. **Shirin Assar:** patient diagnosis, provided clinical data, writing – review and editing. **Mehran Pournazari:** patient diagnosis, provided clinical data, writing – review and editing. **Parviz Soufivand:** patient diagnosis, provided clinical data, writing – review and editing. **Afsaneh Shamsi:** patient diagnosis, provided clinical data, writing – review and editing. **Mahdi Taghadosi:** patient diagnosis, provided clinical data, writing – review and editing. **Alireza Nasirifar:** patient diagnosis, provided clinical data, writing – review and editing. All authors have read and approved the final version of the manuscript for publication.

## Ethics Statement

This study was done in line with the principles of the Declaration of Helsinki. Approval was granted by the Ethics Committee of the Kermanshah University of Medical Sciences (KUMS), Kermanshah, Iran (Ethical code: IR.KUMS.REC.1399.403).

## Consent

Informed consent was obtained from all individual participants included in the study.

## Conflicts of Interest

The authors declare no conflicts of interest.

## Transparency Statement

The lead author Fatemeh Khademi affirms that this manuscript is an honest, accurate, and transparent account of the study being reported; that no important aspects of the study have been omitted; and that any discrepancies from the study as planned (and, if relevant, registered) have been explained.

## Data Availability

The data sets generated during and/or analyzed during the current study are available from the corresponding author upon reasonable request.
